# Tailoring LED spectra and cultivation systems enhances lettuce productivity and nutritional quality in vertical indoor farming

**DOI:** 10.1186/s12870-026-09420-x

**Published:** 2026-07-07

**Authors:** Abdullah Aldiyab, Hayriye Yildiz Dasgan, Boran Ikiz, Sibel Balik, Tugce Temtek, Irem Bicer, Nazim S. Gruda

**Affiliations:** 1 Department of Horticulture, Faculty of Agriculture, University of Cukurova, Adana, 01330 Türkiye; 2 Institute of Plant Sciences and Resource Conservation, Division of Horticultural Sciences, University of Bonn, Bonn, 53113 Germany

**Keywords:** Aeroponics, Ebb and flow, Antioxidants, Controlled-environment agriculture, LED lighting, Multilayer cultivation, Nutritional quality

## Abstract

Light spectrum and cultivation system are major determinants of crop performance in vertical indoor farming and controlled-environment agriculture. Here, we investigated the interaction effects of three LED spectra and two cultivation systems (aeroponic and ebb-and-flow) on the growth, yield, mineral composition, and quality attributes of Batavia lettuce (*Lactuca sativa* L. ‘Capira’) grown under vertical indoor farming conditions. Plants were cultivated under LED1 (70% red, 660 nm + 30% blue, 450 nm), LED2 (full-spectrum PAR, 400–700 nm), and LED3 (65% red, 660 nm + 25% blue, 450 nm + 5% white + 5% far-red, 730 nm) at a standardized light intensity of 200 µmol m⁻² s⁻¹ PPFD using a two-factor experimental design. Significant interaction effects between LED spectrum and cultivation system were observed for plant growth, yield, antioxidant-related compounds, nitrate accumulation, and mineral nutrient composition. Among the evaluated treatments, the LED2 × aeroponic combination resulted in the highest yield (5.02 kg m⁻²), biomass accumulation, and a balanced mineral nutrient profile. In contrast, the LED3 × aeroponic treatment promoted higher total soluble solids and phenolic compound accumulation, whereas the LED1 × Ebb and Flow combination enhanced flavonoid content. The highest vitamin C content and the lowest nitrate accumulation were both observed under the LED1 × aeroponic treatment under the evaluated cultivation conditions. Estimated energy analysis further demonstrated that although LED2 treatments required greater electrical energy input, their higher biomass production resulted in energy-use efficiency values comparable to those obtained under LED3 treatments. These results indicate that coordinated optimization of LED lighting and cultivation system design can improve productivity and quality in multilayer vertical farming systems. However, trade-offs among yield, nitrate accumulation, quality attributes, and estimated energy demand should also be considered. Future research should integrate physiological measurements, direct electrical monitoring, and economic analyses to enable more mechanistic and application-oriented optimization of vertical farming systems.

## Introduction

Controlled environment agriculture (CEA) has become one of the fastest-growing sectors of modern horticulture. It comprises a wide range of production systems, from fully enclosed indoor facilities relying exclusively on artificial lighting, such as vertical farms and plant factories, to advanced greenhouses with or without supplemental lighting, as well as simpler greenhouse structures and high tunnels. CEA enables year-round production, protects crops from adverse climatic conditions, pests, and diseases, and allows precise control of key environmental factors, including temperature, relative humidity, light intensity and spectrum, and CO₂ concentration. As a result, CEA enhances plant growth, product quality, and yield while improving resource-use efficiency and reducing environmental inputs [1–6]. Within controlled environment agriculture systems, light is one of the most critical and precisely managed factors affecting plant performance. It drives photosynthesis, shapes morphological development, and regulates the synthesis of bioactive compounds [6–8]. Key characteristics spectral composition, intensity, and photoperiod—directly influence crop productivity and the sustainability of controlled production systems [9–11]. Among light attributes, intensity and spectral quality exert particularly strong effects on plant physiological responses [12]. Light intensity denotes photon flux output, whereas light quality refers to wavelength composition [13, 14]. Based on wavelength, light is classified as ultraviolet, visible, or infrared; within the visible spectrum, red and blue wavelengths are predominantly used by plants [12] and are the principal drivers of photosynthetic activity and photomorphogenic regulation [15, 16].

Compared with conventional lighting systems, light-emitting diode (LED) technologies are among the most energy-efficient and environmentally sustainable options for controlled environment agriculture (CEA) [6, 17, 18]. In addition to driving photosynthesis, light serves as a key environmental signal that regulates phototropism, seed germination, and flowering [19–21]. Plants also dynamically adjust to fluctuating light conditions by optimizing leaf and chloroplast positioning under low irradiance and activating protective mechanisms, such as chloroplast relocation and pigment synthesis, under excessive light or UV-B exposure [22–24]. These light-mediated responses are fundamental for plant performance in controlled cultivation systems [25–27].

In recent years, LED technologies have been extensively studied in vertical farming and controlled environment agriculture due to their ability to provide precise, crop-specific spectral compositions [11, 28–32]. In systems with limited or no natural light, LEDs allow precise control of light quality to regulate plant growth, morphology, and physiological responses [11, 29, 33, 34]. Red and blue spectra, in particular, strongly influence photosynthetic efficiency, leaf expansion, biomass production, and compact architecture in leafy vegetables such as lettuce [24, 31, 35, 36]. Beyond monochromatic lighting, mixed and full-spectrum LED combinations that better simulate natural light have gained attention for improving not only biomass but also chlorophyll content, antioxidant capacity, and nutritional quality [37–39]. In lettuce, spectra that combine red, blue, white, and far-red wavelengths influence leaf thickness, coloration, nitrate accumulation, and overall market quality, underscoring the need for spectral optimization in vertical farming [36, 40, 41]. However, most studies have examined light spectra in isolation, with limited focus on their interactions with cultivation systems. Thus, the combined effects of LED spectral composition and soilless culture system design on lettuce performance remain insufficiently investigated under fully controlled vertical farming conditions.

While high tunnels are typically soil-based, most CEA operations—particularly vertical farming—rely on soilless culture systems to precisely control the supply of water and nutrients [42–45]. In those systems, plants are grown in nutrient-enriched solutions that create a highly controlled root environment, enabling direct mineral uptake with roots suspended or supported in inert media, thereby enhancing nutrient and water absorption efficiency [46–48]. In these systems, plant performance depends on the interaction between lighting and cultivation technique rather than either factor alone. Accordingly, appropriate selection of light source, spectral quality, and intensity directly affects growth, morphology, and developmental performance [48, 49]. Effective soilless culture operation integrates nutrient solutions, suitable root-supporting media, and artificial lighting, with LED grow lights playing a central role due to their ability to precisely regulate growth and physiological responses by targeting spectral quality and intensity [50–53].

Soilless culture systems deliver nutrient solutions directly to plants and the use of artificial or inert media, allowing precise regulation of nutrient availability and root-zone conditions [54]. This approach enables rapid, year-round production independent of climatic limitations and is particularly advantageous in areas with poor soils or limited arable land. Among soilless culture techniques in controlled environment agriculture, aeroponic and ebb-and-flow systems are widely adopted for their efficiency and adaptability to vertical farming [55, 56]. In aeroponics, roots are suspended in air and intermittently misted with nutrient solution, promoting oxygen exposure, enhanced nutrient uptake, and accelerated biomass accumulation. However, precise technical control is required to avoid root desiccation [57–59]. Conversely, ebb-and-flow systems periodically flood and drain the root zone, alternating nutrient uptake and aeration phases, thereby ensuring stable growth with greater operational simplicity and reliability for commercial-scale production, albeit sometimes with slightly slower growth rates [60–63].

*Lactuca sativa* L. (lettuce) is a commercially important leafy vegetable with high global demand [64]. Owing to its short growth cycle, rapid biomass accumulation, and high market turnover, it is widely cultivated across diverse production systems. It is frequently used as a model species in controlled-environment agriculture research [64–68]. Lettuce is also among the most widely produced crops in soilless culture systems, owing to its shallow root system, minimal structural requirements, and strong responsiveness to environmental manipulation, making it highly suitable for soilless cultivation [66, 69, 70]. Furthermore, lettuce can be efficiently grown in shade structures, greenhouses, and fully enclosed vertical farms, where light, temperature, humidity, and nutrient supply are precisely regulated [71, 72]. Consequently, it is considered an ideal crop for assessing the combined effects of soilless culture system design and artificial lighting strategies on growth, yield, and quality [73–75]. In addition, its high nutritional value, rich in vitamins, minerals, dietary fiber, and bioactive compounds, makes it one of the most consistently demanded fresh vegetables year-round [76–78].

Despite rapid advances in indoor vertical farming and controlled-environment agriculture, optimization of crop performance remains constrained by a limited understanding of how technological components interact within production systems. Previous studies have largely investigated the effects of LED light spectra or soilless culture systems independently. However, in multilayer indoor production environments, plant responses are simultaneously shaped by light quality and root-zone management, and the combined influence of these factors on productivity and nutritional quality remains insufficiently understood. In particular, knowledge gaps remain regarding how specific LED spectral compositions interact with different cultivation system designs to influence growth dynamics, yield formation, antioxidant accumulation, and mineral composition in lettuce under vertical farming conditions. We hypothesized that lettuce performance is governed not only by the individual effects of lighting or cultivation systems, but also by their interaction. We expected that distinct combinations of LED spectral distributions and cultivation system designs would differentially regulate biomass production and quality-related traits, allowing the identification of production strategies that simultaneously optimize yield and nutritional value in indoor farming systems. Therefore, the main objective of this study was to evaluate the combined effects of different LED light spectra and aeroponic and ebb-and-flow systems on lettuce grown in an indoor vertical farming system. In addition, the study aimed to assess antioxidant-related quality and mineral composition under defined system–lighting combinations to identify optimized cultivation configurations for efficient, targeted production in controlled-environment agriculture.

## Materials and methods

**Fig. 1:**
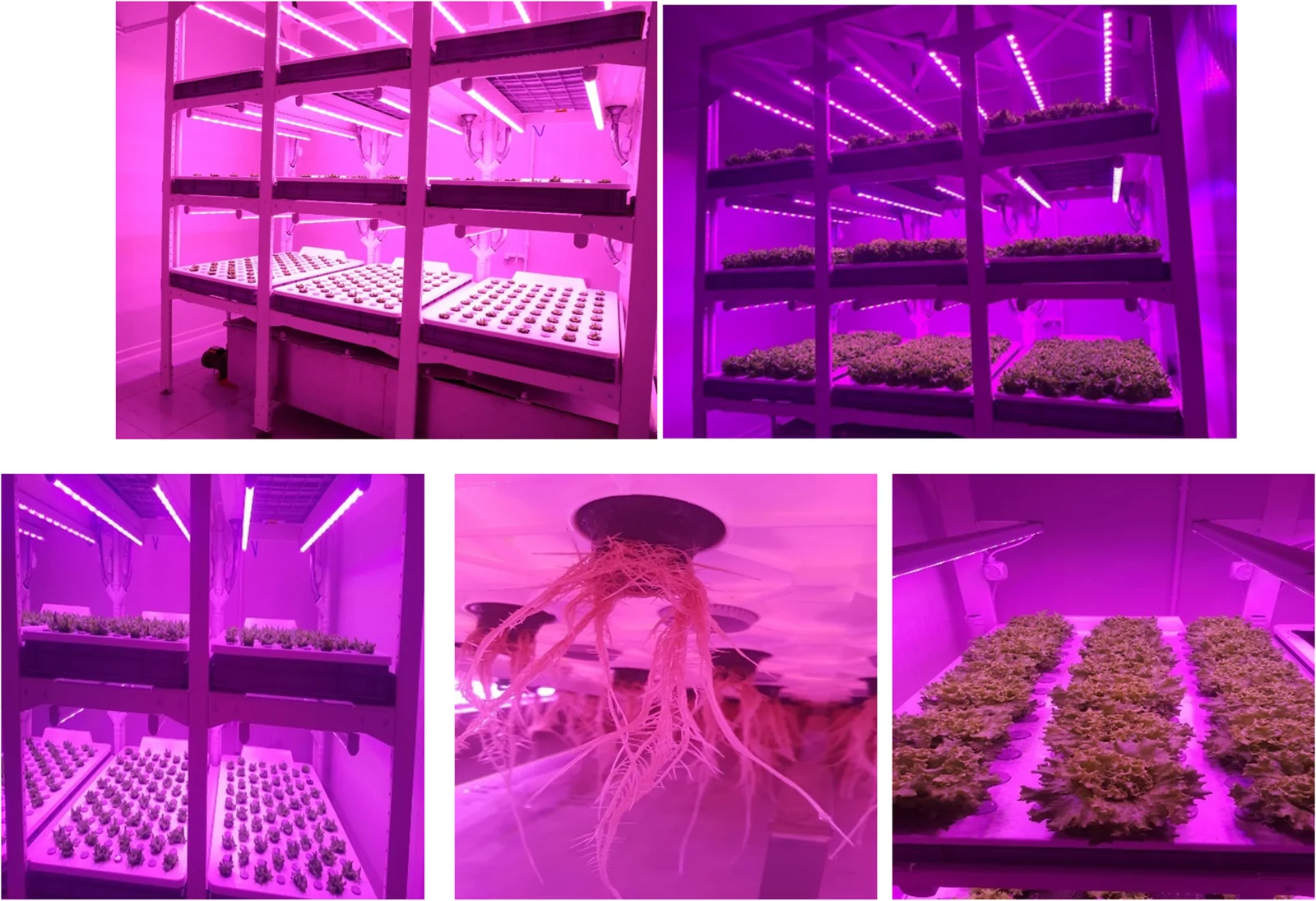
Lettuce plants, grown under different LED light spectra and soilless culture systems

The study was conducted in February 2024 at the “Controlled Vertical Farming R&D Facility” of the Department of Horticulture, Faculty of Agriculture, Cukurova University. The experiment was carried out in fully automated, climate-controlled rooms, each equipped with a three-tier vertical cultivation system and a ceiling height of 3.40 m. The shelves, constructed from galvanized steel, were designed with a 40 cm spacing between tiers (Fig. 1). Each cultivation tier was assigned to one specific LED spectral treatment, thereby establishing three distinct LED spectral combinations within the vertical system. The experiment was conducted using a two-factor factorial design. The first factor consisted of two cultivation systems, aeroponic and ebb-and-flow, while the second factor comprised three different LED light spectra: LED1 with 70% red light (660 nm) and 30% blue light (450 nm); LED2 providing a photosynthetically active radiation (PAR) spectrum in the range of 400–700 nm; and LED3 composed of 65% red (660 nm), 25% blue (450 nm), 5% white light (400–700 nm), and 5% far-red light (730 nm). The experiment included three independent replications, each consisting of a separate cultivation unit containing 28 plants. Lettuce plants were cultivated at a plant density of 28.64 plants m⁻² throughout the experimental period.

### Plant material and growing conditions

Since lettuce is the most widely grown green leafy vegetable in vertical systems in commercial plant factories, a Batavia type, and one of the most prominent in international markets, was selected for this study. Lettuce plants used in this study belonged to the commercial cultivar ‘Caipira’. Seeds were obtained from “AG Tohum” (Türkiye), the official distributor of ‘Enza Zaden’ (Enkhuizen, Netherlands). The plant material used in this study is a commercially cultivated variety and was not collected from wild populations; therefore, no specific permits were required. All experimental procedures complied with institutional and national guidelines. Lettuce seeds were initially germinated in small rockwool blocks under dark conditions in a germination room at 23 °C and 90–95% relative humidity, with radicle emergence occurring within 24–48 h. After reaching the cotyledon stage, seedlings were pre-cultivated for approximately two weeks under visible-spectrum LED lighting (400–700 nm) with a 16/8 h light/dark photoperiod and day/night temperatures of 20/15°C, until they developed 4–5 true leaves and reached a height of 5–7 cm.

Experimental treatments were initiated after the seedlings were transferred to the cultivation systems. Both aeroponic and ebb-and-flow soilless culture systems operated at 50 min intervals throughout the experimental period. In the aeroponic system, nutrient misting was applied for 5 min and 5 s during each cycle. In the ebb-and-flow system, flooding was also maintained for 5 min and 5 s per cycle; however, complete drainage of the cultivation container occurred gradually and required approximately 25 min depending on solution return dynamics. Dissolved oxygen concentrations in the recirculating nutrient solution and root-zone environment were monitored during the experimental period. In the aeroponic system, dissolved oxygen levels in the recirculating nutrient solution ranged between 7.6 and 7.8 ppm, whereas oxygen concentrations within the root misting zone reached 9.7–9.9 ppm. In the ebb-and-flow system, dissolved oxygen concentrations in the recirculating nutrient solution ranged between 7.2 and 7.4 ppm, while values measured during the flooding phase in the root zone ranged between 8.0 and 8.2 ppm.

**Fig. 2:**
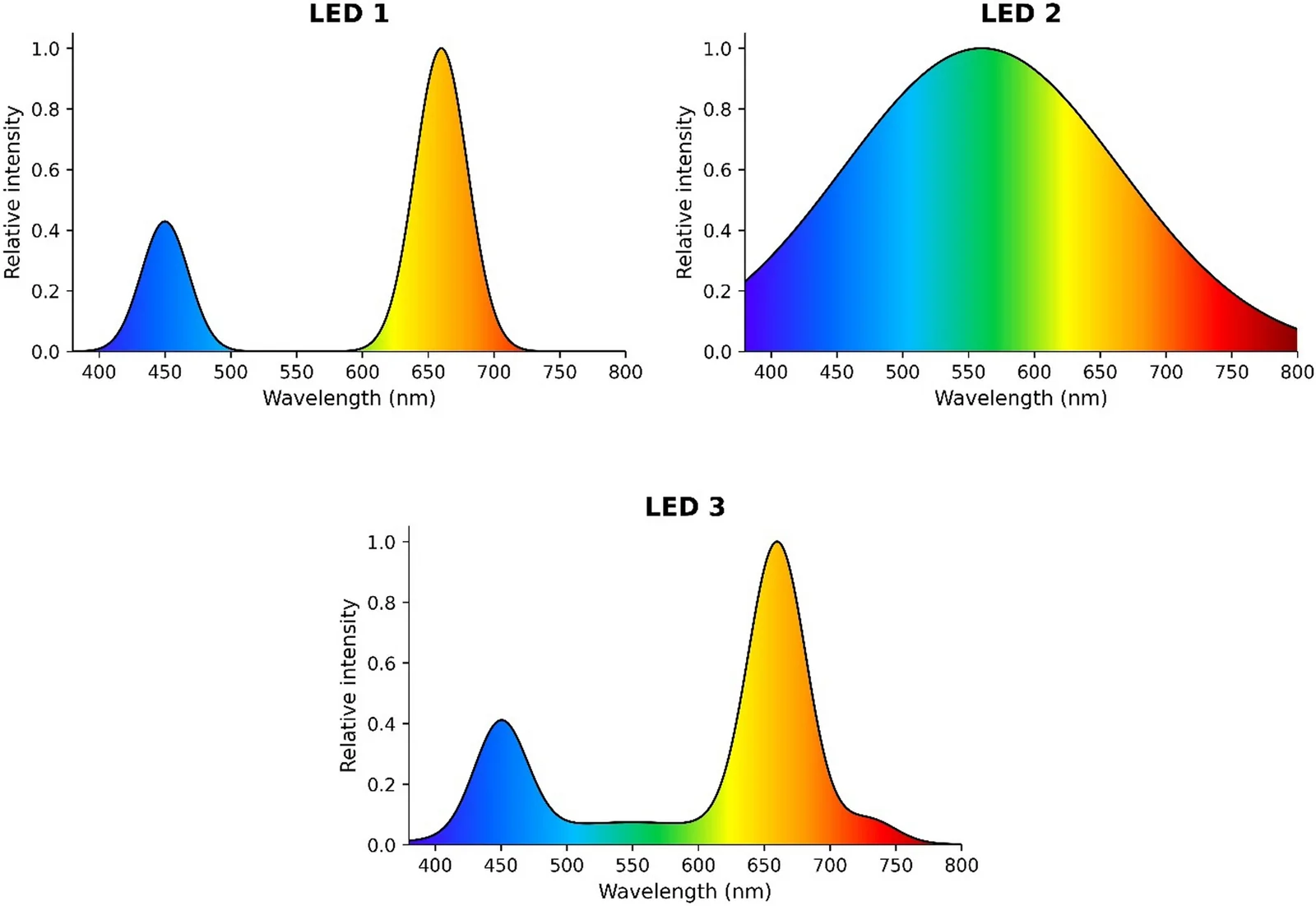
Schematic spectral distributions of the three LED light treatments used in the experiment. LED1 consisted of 70% red light (660 nm) and 30% blue light (450 nm); LED2 represented a full-spectrum photosynthetically active radiation (PAR) range between 400 and 700 nm; and LED3 consisted of 65% red light (660 nm), 25% blue light (450 nm), 5% white light (broad spectrum), and 5% far-red light (730 nm). The spectral curves are presented schematically based on the relative distribution of wavelength components provided for each LED treatment

Following lettuce seedling transplantation, day/night temperatures were maintained at 20/15°C. Light intensity was standardized across all LED treatments and maintained at approximately 200 µmol m⁻² s⁻¹ of photosynthetic photon flux density (PPFD) at the canopy level, as measured with a portable spectral irradiance spectrometer (OHSP350S, HopooColor, China). The distance between the LED panels and the plant canopy was maintained at approximately 26–28 cm, depending on the plant growth stage. A photoperiod of 18 h light and 6 h dark was applied, corresponding to a daily light integral (DLI) of approximately 13.0 mol m⁻² day⁻¹. The schematic spectral distributions of the LED light treatments used in the experiment are presented in Fig. 2.

### Energy consumption and energy use efficiency

The estimated electrical energy demand of each LED treatment was calculated based on the nominal power consumption of the lighting systems, photoperiod duration, and cultivation period. The calculated nominal power values were approximately 140.4 W for LED1, 196.9 W for LED2, and 154.6 W for LED3 under the experimental setup. Energy consumption (kWh) was estimated as:

Energy consumption (kWh) = Rated power (kW) × photoperiod (h day⁻¹) × cultivation period (days).

In addition, energy use efficiency (EUE) was calculated as fresh biomass yield per unit of estimated electrical energy consumption (kg kWh⁻¹). These calculations represent estimated values based on system specifications rather than direct electrical measurements.

### Plant nutrition

The fertigation system consisted of two separate nutrient stock solutions. Stock A was formulated with calcium nitrate and Fe-EDDHA, while Stock B comprised potassium sulfate, mono-potassium phosphate, magnesium sulfate, potassium nitrate, and a micronutrient mix including zinc sulfate, boric acid, manganese sulfate, copper sulfate, and ammonium molybdate. Plant nutrition was provided using a standardized nutrient solution formulated to meet the target concentrations of macro- and micronutrients. Both soilless culture systems operated as closed-loop recirculating systems, in which the nutrient solution was continuously reused throughout the experimental period. The nutrient solution [64] was formulated (mg L⁻¹) to provide nitrogen at 150–220, phosphorus at 30–40, potassium at 270–312, calcium at 170–210, and magnesium at 50–65. Micronutrients were supplied at concentrations of iron at 3.00–5.00, zinc at 0.30–0.55, boron at 0.70–0.97, copper at 0.20–0.30, molybdenum at 0.10–0.20, and manganese at 0.55–0.96. Nutrient solution strength was adjusted according to plant developmental stage throughout the cultivation period. The EC of the nutrient solution was maintained at approximately 1.0 dS m⁻¹ during the early establishment stage, 1.5 dS m⁻¹ during vegetative growth, and 2.0 dS m⁻¹ during the later growth stage. The pH of the nutrient solution was automatically monitored and maintained within the range of 5.7–6.0. Approximately 20% of the recirculating nutrient solution was periodically discharged and replaced with freshly prepared nutrient solution every three weeks throughout the experimental period. Electrical conductivity (EC) and pH were continuously monitored and regulated by an automated control system and additionally checked manually daily.

### Plant harvest

**Fig. 3:**
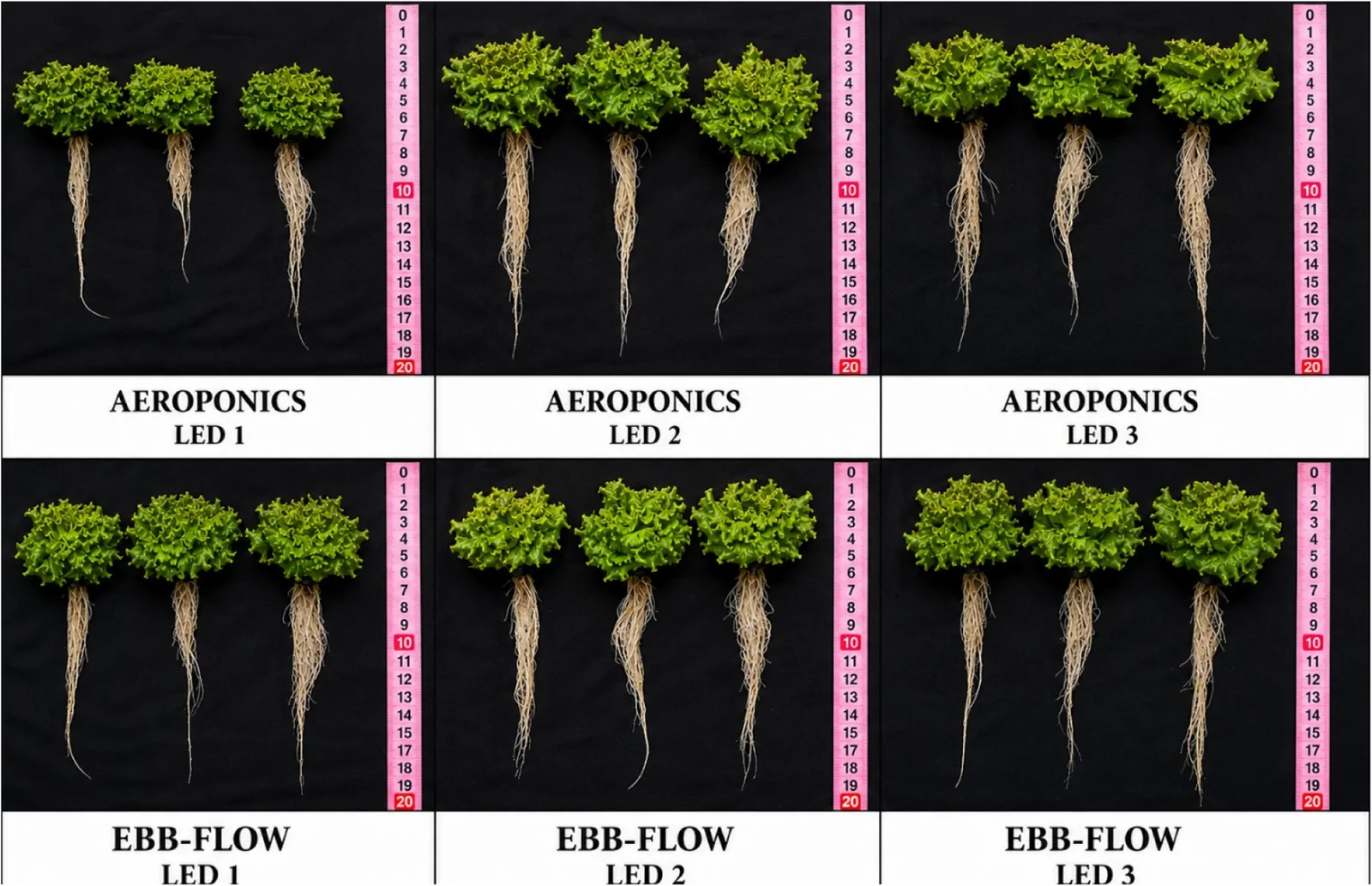
Representative images of lettuce grown under different LED light spectra in aeroponic and ebb–flow soilless culture systems. LED 1: 70% red (660 nm) and 30% blue (450 nm); LED 2: Full-spectrum Photosynthetically Active Radiation (PAR) within the 400–700 nm range; LED 3: 65% red (660 nm), 25% blue (450 nm), 5% white (broad spectrum around 400–700 nm), and 5% far-red (730 nm)

Lettuce seedlings were harvested after 30 days, cultivated under two different cultivation systems and three different LED light spectra (Fig. 3).

### Plant growth parameters and yield

At the end of the cultivation period (30 days), plant height, width, and circumference were measured using a ruler. Stem diameter and length were measured with a digital caliper (Mitutoyo, Tokyo, Japan). The number of leaves per plant was counted directly. Fresh weights of whole plants, leaves, and roots were measured using a digital precision balance with a sensitivity of 0.01 g. For dry weight determination, plant samples were oven-dried at 65 °C for 48 h until a constant weight was reached [79]. Dry matter content was calculated according to the following formula:

$$\text{Dry matter} (\%)=(\mathrm{Dry}\mathrm{weight} / \mathrm{Fresh}\mathrm{weight}) \times\;100$$

Yield was calculated at harvest and expressed as kg m⁻² based on the total fresh weight of plants per unit cultivation area.

### Total soluble solids (TSS), pH, and electrical conductivity (EC) of lettuce

The juice extracted from lettuce was analyzed to determine total soluble solids (TSS), pH, and electrical conductivity (EC). TSS was measured using a digital refractometer (Atago PR-101, Tokyo, Japan). Simultaneously, pH and EC values were determined with a pH/EC meter (WTW pH/Cond 3320, Weilheim, Germany) [80].

### Determination of total phenols

Total phenolic content was determined using a spectrophotometric method modified from Spanos and Wrolstad [81] and Ikiz et al. [82]. For each sample, approximately 5 g of plant tissue was extracted with 80% methanol and ultrasonicated at 40 °C for 10 min to enhance the release of phenolic compounds. The extracts were then centrifuged at 1500 rpm for 5 min. The supernatant was mixed with Folin–Ciocalteu reagent and 20% sodium carbonate, then incubated in the dark for 2 h. Absorbance was measured at 765 nm using a spectrophotometer (Analytik Jena SPECORD 200 PLUS, Jena, Germany). Total phenolic content was quantified using a gallic acid calibration curve and expressed as milligrams of gallic acid equivalents (GAE) per gram of fresh weight (FW) [83].

### Determination of total flavonoids

Total flavonoid content was determined according to the method described by Quettier-Deleu et al. [84] and Ikiz et al. [85]. Briefly, 1 mL of plant extract was mixed with 0.05 mL of 10% aluminum chloride (AlCl₃), 0.05 mL of 1 M potassium acetate, and 1.8 mL of Milli-Q water. The mixture was vortexed thoroughly and incubated in the dark at room temperature for 45 min. Following incubation, absorbance was measured at 450 nm using a spectrophotometer (Analytik Jena SPECORD 200 PLUS, Jena, Germany). Total flavonoid content was expressed as milligrams of rutin equivalents (RE) per 100 g of fresh weight (FW) [83].

### Determination of vitamin C

Vitamin C content was determined using a modified version of the method described by Elgailani and Ishak [86]. Lettuce leaves were first juiced to obtain the plant extract. Five milliliters of the extract were mixed with 45 mL of a 0.4% oxalic acid solution and subsequently filtered. From the resulting solution, 1 mL was mixed with 9 mL of 2,6-dichlorophenolindophenol reagent and thoroughly vortexed. Absorbance was measured at 518 nm using a UV–Vis spectrophotometer [87].

### Determination of nitrate

The colorimetric determination of leaf nitrate accumulation in lettuce leaves was conducted through the transnitration of salicylic acid, as described by Cataldo et al. [88] and modified by Dasgan et al. [80].

### Determination of minerals

The concentrations of potassium (K), magnesium (Mg), calcium (Ca), iron (Fe), manganese (Mn), and zinc (Zn) in lettuce leaves were assessed using an atomic absorption spectrophotometer. Quarters of 3 individual plants from each replication were subjected to a drying process at 65 °C for 48 h, followed by grinding using a mill with a 20-mesh sieve. The resultant leaf powder was combusted in a furnace at 550 °C for 8 h, and the ash was dissolved in 3.3% hydrochloric acid (HCl). Fe, Mn, Zn, and Cu Concentrations were determined through atomic absorption spectrometry in absorbance mode. In contrast, K, Ca, and Mg concentrations were determined in emission mode [89]. The Kjeldahl and Barton methods were used to determine leaf nitrogen and phosphorus levels [90].

### Statistical analyses

The data were analyzed using the JMP statistical software package (version 13.0, SAS Institute Inc., Cary, NC, USA, 2016). Before analysis, data were checked for compliance with the assumptions of analysis of variance, including normality of residuals and homogeneity of variances. A two-factor analysis of variance (ANOVA) was conducted to evaluate the effects of LED light spectrum and soilless culture system, and their interaction, on measured growth, yield, quality, and mineral parameters. When significant treatment effects were detected at the *p* < 0.05 level, treatment means were separated using the Least Significant Difference (LSD) multiple comparison test. Results are presented as mean values accompanied by their respective measures of variability. All statistical evaluations, comparisons, and subsequent interpretations were based on the outcomes of these analyses.

## Results

### Influence of LED lighting and soilless culture systems on lettuce growth and morphology

**Fig. 4:**
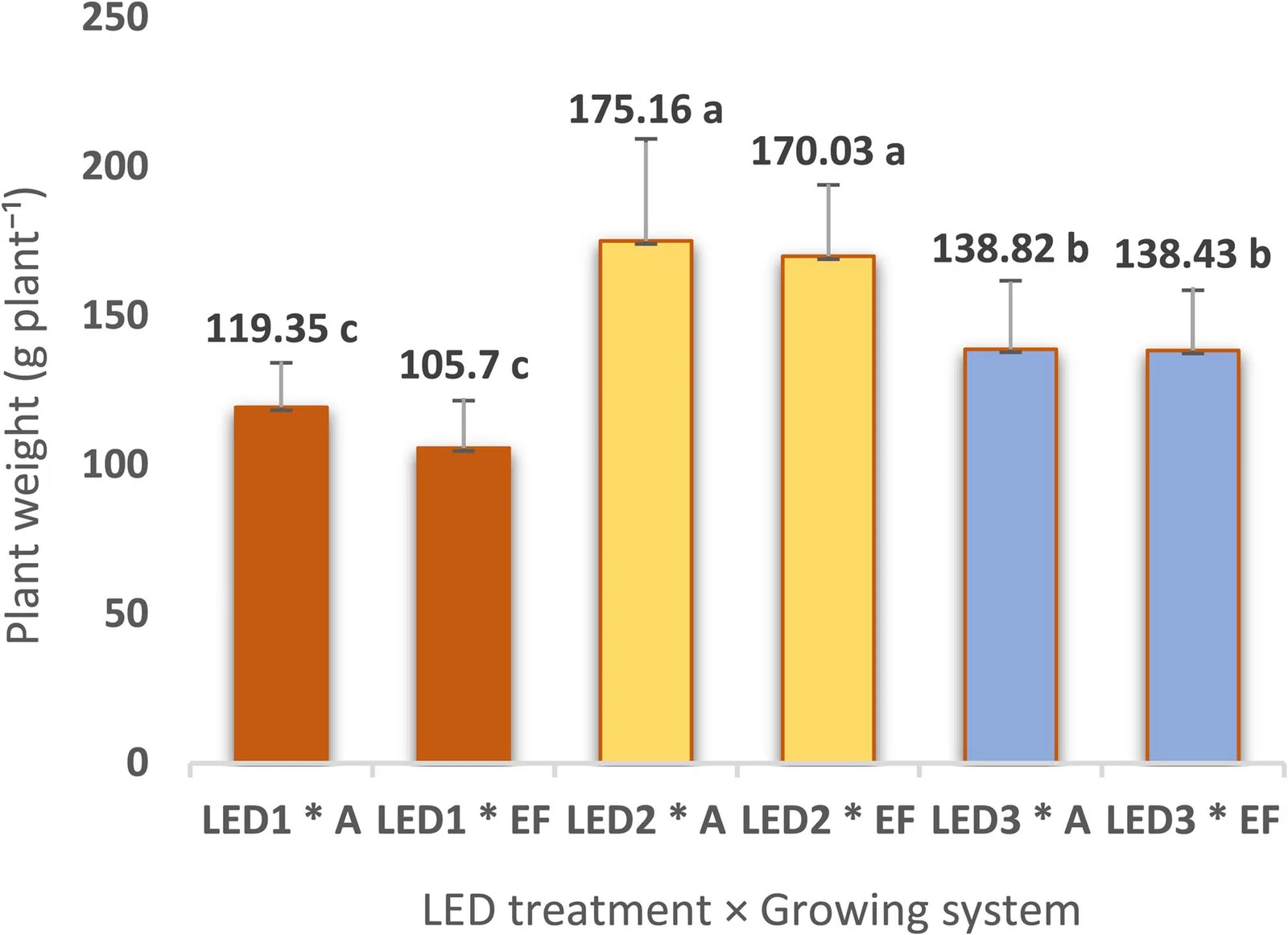
Plant weights of lettuce grown under different LED light spectra in aeroponic and ebb–flow soilless culture systems. LED 1: 70% red (660 nm) and 30% blue (450 nm); LED 2: Full-spectrum Photosynthetically Active Radiation (PAR) within the 400–700 nm range; LED 3: 65% red (660 nm), 25% blue (450 nm), 5% white (broad spectrum around 400–700 nm), and 5% far-red (730 nm). A: Aeroponic. EF: Ebb and Flow. Different letters on the histogram indicate statistically significant differences at *p* < 0.05 according to the LSD test

The interaction between LED spectrum and soilless culture system significantly influenced lettuce plant fresh weight. Overall, LED2 promoted the greatest biomass accumulation in both soilless culture systems, reaching 175.16 g in the aeroponic system and 170.03 g in the ebb-and-flow system. In contrast, plants grown under LED1 exhibited substantially lower fresh weight values, particularly in the ebb-and-flow system. Under LED3, biomass production was similar between aeroponic and ebb-and-flow systems (138.82 and 138.43 g, respectively), indicating a less pronounced system-dependent response than under LED2 (Fig. 4).

**Fig. 5:**
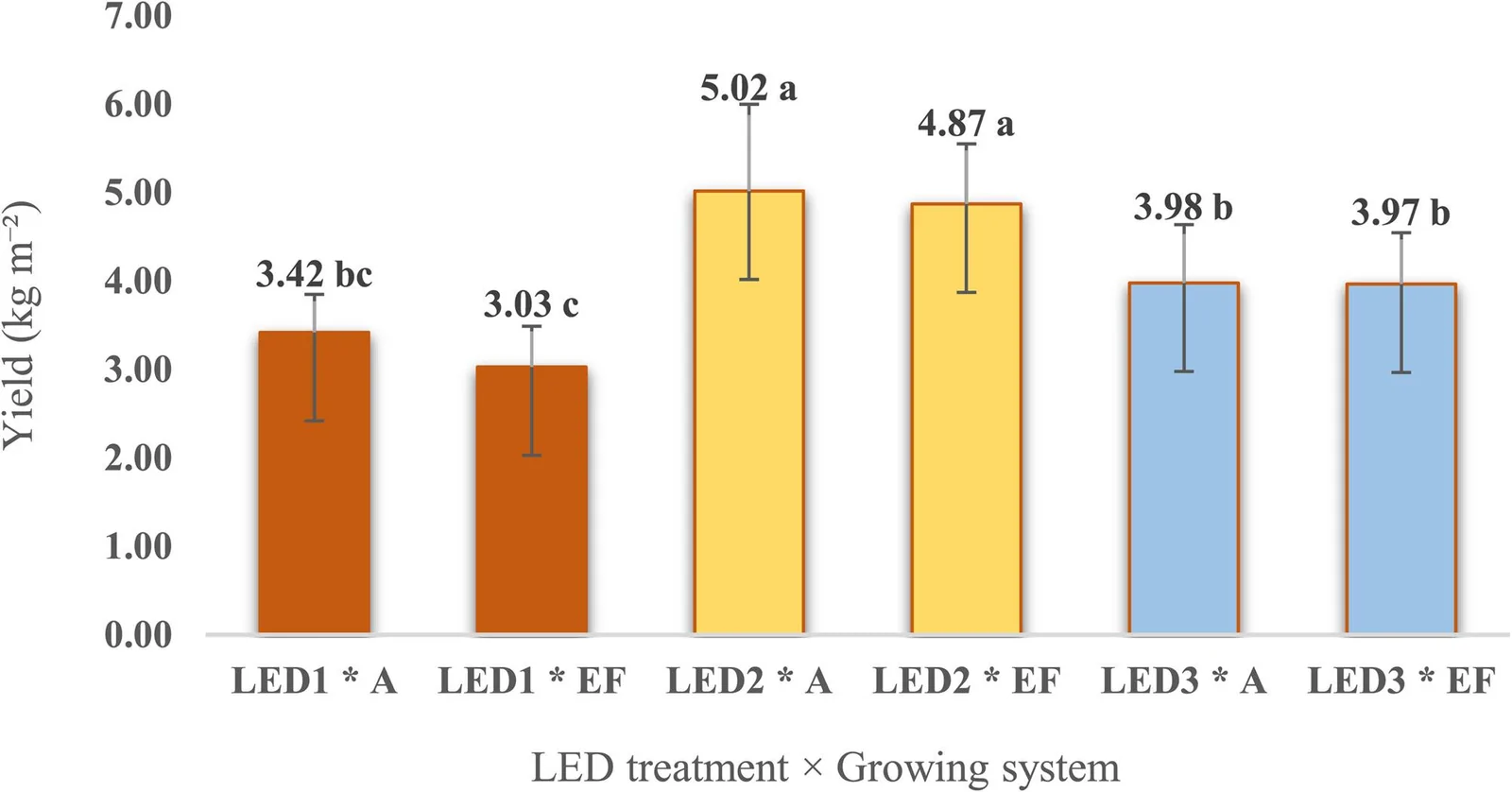
Yield (kg m ²) of lettuce grown under different LED light spectra in aeroponic and ebb–flow soilless culture systems. LED 1: 70% red (660 nm) and 30% blue (450 nm); LED 2: Full-spectrum Photosynthetically Active Radiation (PAR) within the 400–700 nm range; LED 3: 65% red (660 nm), 25% blue (450 nm), 5% white (broad spectrum around 400–700 nm), and 5% far-red (730 nm). A: Aeroponic. EF: Ebb and Flow. Different letters on the histogram indicate statistically significant differences at *p* < 0.05 according to the LSD test

Lettuce yield (kg m⁻²) varied markedly depending on both LED light spectrum and soilless culture system (Fig. 5). The highest yield was recorded under the LED2 × aeroponic system, reaching 5.02 kg m⁻², followed closely by LED2 × ebb and flow with 4.87 kg m⁻². In contrast, lower yields were observed under LED1, particularly in the ebb-and-flow system (3.03 kg m⁻²), whereas the aeroponic counterpart produced 3.42 kg m⁻². Systems involving LED3 resulted in intermediate yields, with comparable values between aeroponic (3.98 kg m⁻²) and ebb and flow (3.97 kg m⁻²) systems.

**Fig. 6:**
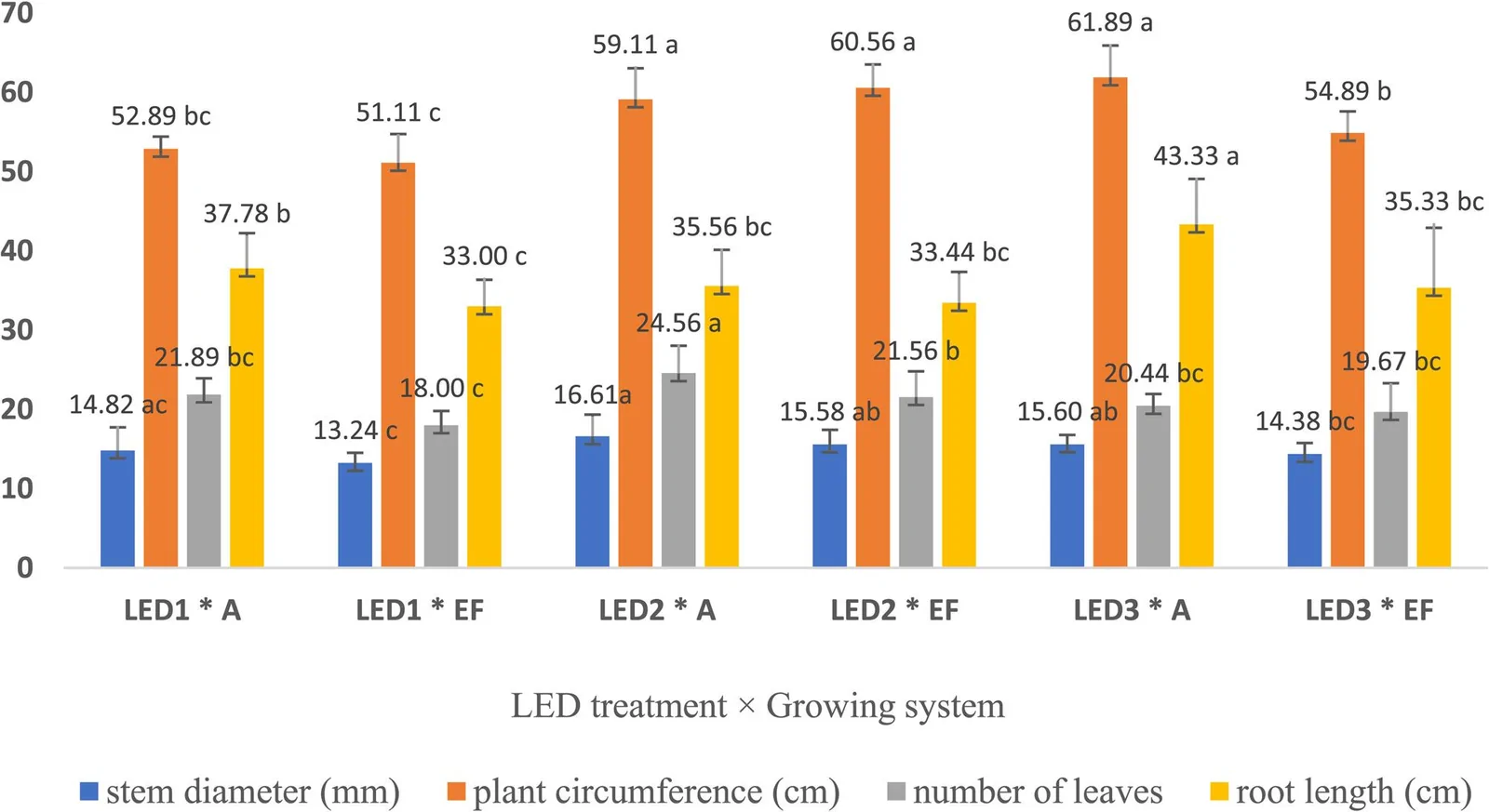
Growth parameters of lettuce cultivated under different LED light spectra in aeroponic and ebb–flow soilless culture systems: stem diameter, plant circumference, number of leaves, and root length. LED 1: 70% red (660 nm) and 30% blue (450 nm); LED 2: Full-spectrum Photosynthetically Active Radiation (PAR) within the 400–700 nm range; LED 3: 65% red (660 nm), 25% blue (450 nm), 5% white (broad spectrum around 400–700 nm), and 5% far-red (730 nm**).** A: Aeroponic. EF: Ebb and Flow. Different letters on the histogram indicate statistically significant differences at *p* < 0.05 according to the LSD test

Morphological parameters were significantly influenced by the interaction between LED spectrum and soilless culture system (Fig. 6). Overall, the aeroponic system promoted greater stem diameter and root development compared with the ebb-and-flow system across most LED treatments. LED2 generally enhanced stem diameter, plant circumference, and leaf number, whereas LED3 was associated with the greatest root length and plant circumference values under aeroponic cultivation. In contrast, LED1 treatments consistently resulted in comparatively lower morphological performance, particularly under ebb-and-flow conditions. The relatively higher root length observed under LED3, especially in the aeroponic system, suggests that far-red supplementation may have promoted vegetative expansion responses and root elongation.

Plant growth parameters were notably affected by the interaction between LED spectrum and soilless culture system (Table 1). Overall, LED2 and LED3 promoted greater canopy development and root growth compared with LED1 in both cultivation systems. In the aeroponic system, LED3 resulted in the greatest plant height and width, whereas LED2 produced the highest root fresh weight and stem dry matter. Similarly, under ebb-and-flow conditions, LED2 generally enhanced canopy expansion and root fresh weight compared with the other LED treatments. In contrast, LED1 consistently resulted in comparatively lower plant width and root biomass values in both soilless culture systems. Root dry matter values remained relatively stable across treatments regardless of cultivation system.

**Table 1:** Effects of three LED light spectra on plant height, plant width, root fresh weight, leaf dry matter, and root dry matter of lettuce grown in two soilless culture systems (Aeroponic and Ebb and Flow)

Soilless Culture System	Plant Height (cm)	Plant Width (cm)	Root Fresh Weight (g)	Leaf Dry Matter (%)	Root Dry Matter (%)
Aeroponic	13.22	20.92	14.47	5.33	4.33
Ebb and Flow	13.03	20.33	14.56	5.11	4.20
LSD_0.05_	NS	NS	NS	NS	NS
*P value*	0.2544	0.1075	0.8869	0.2471	0.2188
Lighting					
LED1	12.61 b	18.77 c	10.91 c	5.33	4.20
LED2	13.33 a	22.05 a	17.86 a	5.31	4.29
LED3	13.44 a	21.05 b	14.77 b	5.02	4.29
LSD_0.05_	0.3961	0.889	1.576	NS	NS
*P value*	0.0002	< 0.0001	< 0.0001	0.3609	0.7071
Soilless culture System × Lighting					
LED1**×** Aeroponic	12.77 bc	19.11 bc	11.01 c	5.22 ab	4.23
LED1**×** Ebb and Flow	12.44 c	18.44 c	10.81 c	5.44 ab	4.18
LED2**×** Aeroponic	13.22 ab	21.66 a	17.90 a	5.65 a	4.18
LED2**×** Ebb and Flow	13.44 a	22.44 a	17.83 a	4.97 ab	4.40
LED3**×** Aeroponic	13.66 a	22.00 a	14.49 b	5.13 ab	4.19
LED3**×** Ebb and Flow	13.22 ab	20.11 b	15.05 b	4.91 b	4.4
LSD_0.05_	0.5598	1.259	2.253	0.683	NS
*p value*	0.2034	0.0161	0.8757	0.1819	0.4713

LED 1: 70% red (660 nm) and 30% blue (450 nm); LED 2: Full-spectrum Photosynthetically Active Radiation (PAR) within the 400–700 nm range; LED 3: 65% red (660 nm), 25% blue (450 nm), 5% white (broad spectrum around 400–700 nm), and 5% far-red (730 nm). Means followed by different letters in the same column differ significantly at p < 0.05 (LSD test)

*NS* Not significant

### Influence of LED Lighting and Soilless Culture Systems on Chemical Composition and Quality Parameters

The interaction between soilless culture system and LED lighting had significant effects on pH, electrical conductivity (EC), total soluble solids (TSS), total phenolic content, flavonoid content, vitamin C, and nitrate accumulation in lettuce (Table 2).

**Table 2:** Effects of three LED light spectra on pH, electrical conductivity (EC), total soluble solids (TSS), total phenolic content, flavonoid content, vitamin C, and nitrate levels of lettuce grown in two soilless culture systems

Soilless Culture System	pH	EC (dS/m)	TSS (°Brix)	Total Phenolics (mg GA/100 g FW)	Total Flavonoids (mg RU/100 g FW)	Vitamin C (mg/100 g FW)	Nitrate (mg/kg FW)
Aeroponic	6.13 a	9.12 b	1.19 a	51.27 a	22.10 a	37.89 a	717.33 a
Ebb and Flow	6.08 b	9.43 b	1.13 a	50.34 b	21.35 a	37.13 a	655.11 a
LSD_0.05_	0.018	NS	NS	0.63	NS	NS	NS
*P value*	< 0.0001	0.0001	0.5462	0.0086	0.1181	0.0933	0.1804
Lighting							
LED1	6.15 a	9.19 b	1.14 ab	49.71 c	22.26 a	37.97 a	537.66 b
LED2	6.07 b	8.96 c	1.05 b	51.81 a	21.64 a	37.93 a	647.16 b
LED3	6.09 b	9.68 a	1.29 a	50.90 b	21.27 a	36.64 b	873.83 a
LSD_0.05_	0.022	0.164	0.249	0.773	NS	1.180	117.88
*P value*	< 0.0001	< 0.0001	0.1289	0.0005	0.2226	0.0401	0.0003
Soilless culture System × Lighting							
LED1× Aeroponic	6.16 a	8.57 d	1.06 b	49.86 bc	21.10 bc	38.89 a	488.66 d
LED1× Ebb and Flow	6.14 a	9.81 a	1.23 b	49.56 cd	23.43 a	37.04 b	586.66 cd
LED2× Aeroponic	6.09 b	8.95 c	0.91 b	50.78 b	22.68 ab	38.87 a	744.33 bc
LED2× Ebb and Flow	6.05 c	8.97 c	1.19 b	52.83 a	20.60 c	36.99 b	550.00 d
LED3× Aeroponic	6.14 a	9.84 a	1.60 a	53.16 a	22.52 ab	35.92 b	919.33 a
LED3**×** Ebb and Flow	6.04 c	9.51 b	0.99 b	48.63 d	20.03 c	37.35 ab	828.66 ab
LSD_0.05_	0.031	0.231	0.352	1.093	1.687	1.587	165.58
*p value*	0.0731	< 0.0001	0.0022	< 0.0001	0.0019	0.0118	0.0553

LED 1: 70% red (660 nm) and 30% blue (450 nm); LED 2: Full-spectrum Photosynthetically Active Radiation (PAR) within the 400–700 nm range; LED 3: 65% red (660 nm), 25% blue (450 nm), 5% white (broad spectrum around 400–700 nm), and 5% far-red (730 nm). EC Electrical Conductivity, TSS Total Soluble Solids. Means followed by different letters in the same column differ significantly at *p* < 0.05 (LSD test)

*NS* Not significant, *FW* Fresh weight, *GA* Gallic acid, *RU* Rutin

Quality parameters were significantly influenced by the interaction between the soilless culture system and the LED spectrum (Table 2). The responses of quality traits to LED treatments differed markedly between aeroponic and ebb-and-flow systems. Under aeroponic cultivation, LED3 substantially increased EC, TSS, total phenolic content, and nitrate accumulation compared with the other LED treatments, while vitamin C content was higher under LED1 and LED2. In contrast, under ebb-and-flow conditions, LED2 promoted the highest phenolic content, whereas LED1 resulted in the highest flavonoid concentration and LED3 produced the highest vitamin C and nitrate levels. Nitrate accumulation exhibited one of the strongest interaction responses, reaching 919.33 mg/kg under the LED3 × aeroponic combination and 828.66 mg/kg under the LED3 × ebb-and-flow combination. In contrast, considerably lower nitrate concentrations were recorded under LED1 and LED2 treatments. These findings indicate that the influence of LED spectral composition on lettuce quality attributes was strongly dependent on the soilless culture system applied. 

### Influence of LED Lighting and Soilless culture Systems on Mineral Content

Mineral nutrient composition was significantly influenced by the interaction between LED spectrum and soilless culture system (Table 3). Overall, aeroponic cultivation promoted higher macro- and micronutrient accumulation than ebb-and-flow conditions. Nitrogen and phosphorus concentrations were highest under the LED1 × aeroponic treatment, whereas potassium accumulation was greatest under LED3 × aeroponic conditions. Magnesium content also showed a strong response to cultivation system, reaching its maximum value under LED1 × aeroponic treatment, while the lowest Mg concentration was recorded under LED1 × ebb-and-flow conditions. Calcium accumulation was markedly enhanced under LED2 × aeroponic treatment, indicating a positive interaction between full-spectrum lighting and aeroponic cultivation for Ca uptake.

**Table 3:** Macro (%) and micro (mg kg^− 1^) mineral nutrient concentrations on a dry weight basis of lettuce grown under three different LED lights and two soilless culture systems

Soilless culture System	N	P	K	Mg	Ca	Fe	Mn	Cu	Zn
Aeroponic	5.35 a	0.33 a	9.01 a	0.85 a	2.19 a	71.75 a	18.91 b	11.00 a	46.41 a
Ebb and Flow	5.06 b	0.32 a	8.30 b	0.77 b	1.87 b	74.75 a	21.75 a	11.16 a	36.41 b
LSD_0.05_	0.089	NS	0.392	0.015	0.151	NS	1.960	NS	2.771
*P value*	< 0.0001	0.1762	0.0016	< 0.0001	0.0005	0.3213	0.008	0.6997	< 0.0001
Lighting									
LED1	5.45 a	0.34 a	8.00 b	0.75 c	2.05 ab	64.25 b	20.25ab	11.37 a	41.50 a
LED2	5.15 b	0.32 a	8.45 a	0.81 b	2.13 a	87.12 a	21.87 a	10.25 a	42.62 a
LED3	5.01 c	0.33 a	9.13 a	0.86 a	1.92 b	68.50 b	18.87 b	11.62 a	40.12 a
LSD_0.05_	0.109	NS	0.481	0.018	0.185	7.843	2.421	NS	NS
*P value*	< 0.0001	0.1599	0.0004	< 0.0001	0.0774	< 0.0001	0.0569	0.041	0.3221
Soilless culture System × Lighting									
LED1 × Aeroponic	5.47 a	0.37 a	8.85 a	0.91 a	1.76 b	56.25 c	20.00 b	11.75 a	45.25 a
LED1 × Ebb and Flow	5.43 a	0.31 b	7.14 b	0.60 d	2.33 a	72.25 b	20.50 b	11.00 a	37.75 b
LED2 × Aeroponic	5.24 b	0.32 b	8.88 a	0.80 c	2.49 a	90.25 a	18.50 b	9.25 b	47.50 a
LED2 × Ebb and Flow	5.06 c	0.32 b	8.82 a	0.81 bc	1.77 b	84.00 a	25.25 a	11.25 a	37.75 b
LED3 × Aeroponic	5.34 ab	0.31 b	9.13 a	0.83 b	2.31 a	68.75 b	18.25 b	12.00 a	46.50 a
LED3 × Ebb and Flow	4.69 d	0.31 b	8.95 a	0.89 a	1.52 b	68.25 b	19.50 b	11.25 a	33.75 b
LSD_0.05_	0.153	0.027	0.679	0.026	0.262	11.083	3.425	1.564	4.816
*p value*	< 0.0001	0.0027	0.0052	< 0.0001	< 0.0001	0.0227	0.0293	0.0264	0.2883

LED 1: 70% red (660 nm) and 30% blue (450 nm); LED 2: Full-spectrum Photosynthetically Active Radiation (PAR) within the 400–700 nm range; LED 3: 65% red (660 nm), 25% blue (450 nm), 5% white (broad spectrum around 400–700 nm), and 5% far-red (730 nm). Means followed by different letters in the same column differ significantly at p < 0.05 (LSD test)

*NS* Not significant

Among the micronutrients, LED2 treatments generally promoted greater accumulation of Fe and Mn, particularly under aeroponic conditions. The highest Fe concentrations were recorded under LED2 in both soilless culture systems, whereas Mn reached its maximum level under the LED2 × ebb-and-flow combination. Zinc accumulation was consistently higher in aeroponically grown plants across all LED treatments, whereas Cu concentrations showed comparatively smaller treatment-dependent variation. These findings indicate that mineral nutrient uptake and accumulation responses were strongly dependent on the combined effects of LED spectral composition and soilless culture system. 

### Estimated energy consumption and energy use efficiency

Estimated energy consumption and energy use efficiency (EUE) differed among LED spectral treatments due to variations in calculated nominal power demand (Table 4). Among the evaluated treatments, LED2 exhibited the highest estimated energy consumption under both hydroponic systems, whereas LED1 showed the lowest energy demand. The highest lettuce yield was recorded under the LED2 × aeroponic treatment (5.02 kg m⁻²), while the greatest EUE values were observed under LED3 treatments in both aeroponic and ebb-and-flow systems. Overall, EUE values ranged from 0.0391 to 0.0466 kg kWh⁻¹ across treatments.

**Table 4:** Estimated energy consumption and energy use efficiency (EUE) of lettuce grown under different LED spectral treatments and soilless culture systems

Soilless culture System × Lighting	Lettuce Yield (kg m⁻²)	Energy (kWh m⁻²)	EUE (kg kWh⁻¹)
LED1 **×** Aeroponic	3.42	77.56	0.0441
LED1 **×** Ebb and Flow	3.03	77.56	0.0391
LED2 **×** Aeroponic	5.02	108.79	0.0461
LED2 **×** Ebb and Flow	4.87	108.79	0.0448
LED3 **×** Aeroponic	3.98	85.41	0.0466
LED3 **×** Ebb and Flow	3.97	85.41	0.0465

LED 1: 70% red (660 nm) and 30% blue (450 nm); LED 2: Full-spectrum Photosynthetically Active Radiation (PAR) within the 400–700 nm range; LED 3: 65% red (660 nm), 25% blue (450 nm), 5% white (broad spectrum around 400–700 nm), and 5% far-red (730 nm)

## Discussion

This study evaluated the effects of two cultivation systems and three LED light spectra on growth parameters, quality traits, and mineral nutrient uptake of lettuce grown in a controlled-environment vertical farming system. Both the LED light spectrum and the soilless culture system type were key determinants of plant performance, leading to significant differences across the evaluated parameters.

### Influence of LED lighting and soilless culture systems on lettuce growth and morphology

The highest biomass accumulation was obtained under the LED2 treatment in both soilless culture systems, particularly under aeroponic cultivation. The enhanced growth response observed under LED2 may be associated with the broader spectral distribution of this treatment, which likely supported more balanced canopy development, light interception, and biomass production compared with the narrower-spectrum LED1 treatment. In contrast, the comparatively lower plant weight recorded under LED1, particularly in the ebb-and-flow system, may reflect growth limitations commonly associated with high-blue-light, narrow-spectrum environments. These findings are consistent with previous studies reporting that broad-spectrum LEDs more closely resembling natural daylight can improve biomass accumulation and plant growth performance compared with narrow-spectrum lighting conditions [91, 92].

Morphological responses also varied markedly depending on the interaction between LED spectrum and soilless culture system. The LED2 × aeroponic combination promoted the greatest stem diameter and leaf number, indicating a strong positive effect on vegetative growth and canopy formation. In contrast, the LED3 × aeroponic treatment produced the highest plant circumference and root length, suggesting that far-red supplementation may have enhanced responses to vegetative expansion and root elongation. The stronger morphological responses generally observed under aeroponic cultivation may additionally be associated with improved root-zone oxygen availability and nutrient uptake efficiency provided by the aeroponic interface.

These findings indicate that the aeroponic system generally promoted morphological enhancement, likely due to improved root-zone oxygen availability and more efficient nutrient delivery directly to the root surface. In aeroponic systems, roots remain suspended in air while receiving nutrient mist applications, which can enhance oxygen diffusion, nutrient uptake efficiency, and root activity compared with substrate-based or intermittently flooded systems [56, 59, 63]. The greater root length and improved canopy-related growth parameters observed under aeroponic treatments in the present study may therefore be associated with the favorable root-zone environment provided by the aeroponic interface.

Although blue light plays an important role in regulating photosynthesis, stomatal activity, and morphophysiological responses, excessively high proportions of blue light under narrow-spectrum LED conditions may restrict leaf expansion and biomass accumulation. In the present study, LED1 had the highest proportion of blue light (30%) and resulted in lower plant biomass than LED2. This response may be associated with the compact growth patterns and reduced canopy expansion commonly reported under high-blue spectral conditions, often referred to as “blue light syndrome” in controlled-environment cultivation systems [93, 94]. Previous studies have demonstrated that different spectral regions can distinctly influence plant growth and morphophysiological responses. Fujiuchi and Fujiwara [95] reported that UV, blue, and far-red light exposure to the root zone may suppress shoot growth. In contrast, Lin et al. [96] demonstrated that combined red and blue light can improve morphological quality. Similarly, several studies have emphasized that different regions within photosynthetically active radiation (400–700 nm) regulate plant development differently and that the red-to-blue light ratio plays an important role in determining growth responses [97–99]. Wang et al. [100] further reported that increasing the red-to-blue ratio may enhance seedling growth parameters while simultaneously reducing chlorophyll accumulation. In the present study, the superior biomass and canopy development observed under LED2 suggest that broader-spectrum lighting conditions may have supported more balanced vegetative growth responses compared with the narrower spectral compositions of LED1 and LED3.

Previous studies have shown that spectral composition strongly influences biomass accumulation and morphological development in lettuce cultivation. Ju et al. [101] demonstrated that broad-spectrum lighting supplemented with red and far-red wavelengths can enhance biomass production. However, far-red illumination may simultaneously reduce chlorophyll and nitrogen concentrations, as also reported by Li and Kubota [102] and Vaštakaitė et al. [103]. These findings suggest that plant responses are strongly dependent on both spectral balance and wavelength interactions. Similarly, Matysiak et al. [104] reported that specific red-light combinations enhanced lettuce biomass production, leaf number, and canopy-related growth traits, although different spectral ratios elicited distinct morphological responses. Comparable treatment-dependent variations in canopy development and root morphology were also observed in the present study under different LED × soilless culture system combinations.

The findings of the present study demonstrate that aeroponic cultivation provided clear advantages for biomass accumulation and morphological development compared with the ebb-and-flow system. The stronger growth responses observed under aeroponic conditions may be associated with improved root-zone oxygen availability and more efficient nutrient delivery directly to the root surface via fine nutrient mist. During the experimental period, dissolved oxygen concentrations in the recirculating nutrient solution ranged between 7.6 and 7.8 ppm in the aeroponic system and 7.2–7.4 ppm in the ebb-and-flow system. Furthermore, oxygen concentrations measured directly within the aeroponic root misting zone reached 9.7–9.9 ppm, whereas values measured during the flooding phase of the ebb-and-flow system remained lower (8.0–8.2 ppm). Enhanced oxygen availability in the aeroponic root zone may have promoted greater nutrient uptake efficiency, root metabolic activity, and vegetative growth performance [57–59]. In particular, the combination of LED2 with aeroponic cultivation consistently resulted in superior plant biomass and canopy development, indicating a strong interaction between spectral quality and root-zone environmental conditions.

Among the evaluated LED treatments, the highest lettuce yield per unit area was obtained under LED2, which provided a broader photosynthetically active radiation spectrum (400–700 nm) than the narrower spectra of LED1 and LED3. Previous studies have frequently reported that red and blue wavelength combinations promote plant growth and biomass accumulation through their complementary physiological functions [105–107]. However, the superior performance of LED2 in the present study suggests that broader-spectrum lighting conditions may provide additional advantages under vertical farming environments by supporting more balanced canopy development, light interception, and biomass partitioning. The inclusion of intermediate wavelengths within the PAR region may have contributed to improved coordination of plant growth responses and more efficient utilization of available light energy under controlled-environment conditions.

### Influence of LED lighting and soilless culture systems on chemical composition and quality parameters

The total soluble solids (TSS) content was highest in the aeroponic system under the LED3 treatment. Similarly, the maximum total phenolic content was recorded in the LED3 × aeroponic combination. Regarding flavonoid content, the highest values were observed under LED1 treatment in the ebb-and-flow system. Although no pronounced differences in vitamin C content were observed among treatments, relatively higher values were detected under LED1 × aeroponic conditions. Regarding nitrate content, the lowest levels were observed in the LED1 × aeroponic and LED2 × ebb-and-flow treatments. Overall, these findings indicate that both the light spectrum and the type of soilless culture system exert interactive and selective effects on plant quality parameters. Several studies have suggested that red light wavelengths may enhance phenolic compound accumulation. For instance, Li and Kubota [102] and Samuoliene et al. [108] reported increases in phenolic concentrations of 28.5% in red baby lettuce and 6% in green lettuce, respectively, under red light exposure. In contrast, Sawatdee et al. [38] reported that red light was the least effective treatment for stimulating phenolic accumulation. In their study, the total concentration of detected phenolic compounds was highest under the Blue treatment, followed by the control, Green, and Red treatments, respectively. Flavonoids and phenolic compounds are recognized for their anti-inflammatory, antioxidant, and disease-preventive properties [109]. Previous studies have shown that light quality strongly influences their accumulation, with white and blue light promoting higher levels of phenolics and flavonoids compared to red or red–blue light treatments [39, 110].

Regarding nitrate accumulation, the findings align with previous studies showing that LED spectra influence nitrate metabolism. Knyazeva et al. [111] demonstrated that nighttime blue light substantially reduced nitrate content in certain cultivars compared with red light, whereas in the pre-harvest phase, red light more effectively limited nitrate accumulation than blue light. Similarly, Mirgos et al. [112], Ohashi-Kaneko et al. [113], Lillo [114], and Signore et al. [115] reported that blue and red wavelengths can decrease nitrate concentrations. These studies collectively indicate that nitrate dynamics depend on both the timing and spectral composition of light.

Given that nitrate accumulation is strongly influenced by both fertilizer management and light spectral composition, the strategic selection of LED spectra may help regulate nitrate levels in leafy vegetables. However, the present results also demonstrate that nitrate responses can vary with the specific spectral combination used, particularly under far-red supplementation. The increased nitrate accumulation observed under the LED3 treatment may be associated with changes in plant growth allocation patterns induced by far-red supplementation. Far-red light is known to promote vegetative expansion responses, including increased canopy development and leaf expansion, which may alter the balance between nitrate uptake and nitrate assimilation processes [8, 101]. In the present study, the higher plant circumference, plant width, and root length recorded under LED3 may indicate enhanced growth expansion, potentially leading to greater nitrate retention within leaf tissues. However, because nitrate reductase activity and related nitrogen assimilation mechanisms were not directly evaluated, these interpretations should be approached with caution. Nevertheless, although nitrate concentrations increased under the LED3 treatment, the measured values remained below the maximum nitrate limits reported for lettuce under European regulations [64].

When compared with the quality parameters reported by Matysiak et al. [104] and Gómez and Jiménez [116], the present findings further demonstrate that lettuce quality is strongly influenced by the interaction between LED spectrum and soilless culture system. In the current study, the LED3 × aeroponic combination yielded the highest TSS, phenolic content, and EC, whereas the LED1 × ebb-and-flow treatment resulted in the highest acidity and flavonoid accumulation. These responses indicate that quality-related traits are not regulated solely by spectral composition, but are also strongly dependent on root-zone conditions and soilless culture system characteristics.

The results suggest that the combined optimization of LED spectral composition and soilless culture strategy may be an effective approach to improving lettuce quality attributes, biochemical composition, and nitrate management in controlled-environment agricultural systems.

### Influence of LED lighting and soilless culture systems on mineral content

The interaction between LED spectrum and soilless culture system strongly influenced mineral nutrient composition. Overall, aeroponic cultivation generally promoted greater accumulation of several macroelements, particularly N, K, Mg, Ca, and Zn. In contrast, the ebb-and-flow system tended to support higher Fe and Mn concentrations under specific LED treatments. Among the evaluated lighting conditions, LED2 generally increased Ca and Fe accumulation, whereas LED3 increased K concentrations. The combined LED2 × aeroponic treatment produced the most balanced mineral nutrient profile, characterized by high concentrations of Ca, Fe, Zn, K, and Mg under controlled-environment conditions.

Nitrogen accumulation varied considerably among treatments, with the highest concentration observed under LED1 × aeroponic conditions and the lowest under LED3 × ebb-and-flow conditions, indicating a strong interaction effect between spectral composition and cultivation system on N uptake. Similarly, potassium and magnesium concentrations were generally greater under aeroponic cultivation, particularly under LED3 and LED1 treatments, respectively. Calcium accumulation was markedly enhanced under the LED2 × aeroponic combination, further supporting the positive influence of full-spectrum lighting combined with improved root-zone conditions on mineral uptake efficiency.

The observed mineral nutrient responses are consistent with previous reports indicating that LED spectral composition can significantly influence nutrient accumulation patterns in lettuce [33]. In addition, the improved nutrient acquisition observed under aeroponic cultivation agrees with findings reported by Chowdhury et al. [67], who demonstrated greater nutrient uptake efficiency under aeroponic conditions. The stronger mineral accumulation responses observed under aeroponic cultivation in the present study may be associated with enhanced root-zone oxygen availability and more efficient nutrient absorption dynamics compared with the ebb-and-flow system.

The findings demonstrate that different LED × soilless culture system combinations selectively promoted specific mineral nutrients. LED1 × aeroponic conditions favored nitrogen and magnesium accumulation; LED3 × aeroponic conditions enhanced potassium uptake, whereas LED2 × aeroponic conditions maximized calcium, iron, and zinc concentrations. These results further confirm that mineral nutrient acquisition in lettuce is jointly regulated by both spectral quality and soilless culture system design under controlled-environment agriculture conditions.

The observed improvements in plant growth, yield, quality traits, and mineral nutrient accumulation under specific LED × soilless culture system combinations may therefore be associated with differences in root-zone environment, nutrient uptake efficiency, and plant growth responses under varying spectral conditions. However, because detailed physiological and biochemical mechanisms were not directly evaluated in the present study, these interpretations should be approached with caution.

### Estimated energy consumption and energy use efficiency

The estimated energy analysis demonstrated that although LED2 promoted the highest biomass accumulation and yield, it also required greater electrical energy input compared with LED1 and LED3 treatments. Nevertheless, the EUE values obtained under LED2 and LED3 were relatively similar, indicating that the increased productivity achieved under LED2 partially compensated for its greater energy demand. In contrast, LED3 treatments exhibited slightly greater EUE values under both hydroponic systems, suggesting that mixed-spectrum lighting including far-red wavelengths may provide a comparatively balanced relationship between biomass production and estimated energy consumption under controlled-environment conditions.

## Conclusions

The interaction between LED light spectrum and cultivation system influenced lettuce performance in indoor vertical farming systems. Significant effects were observed for growth, yield, quality attributes, mineral nutrient composition, estimated energy consumption, and energy use efficiency (EUE), indicating that plant responses depended on the combined effects of canopy light environment and root-zone conditions rather than on individual factors alone. Among the evaluated treatments, the LED2 × aeroponic combination generally resulted in the highest biomass accumulation, yield, canopy development, and a balanced mineral nutrient profile. The results further showed that lettuce quality attributes responded differentially to LED spectral composition. While LED2 and LED3 enhanced several growth and quality-related parameters, LED3 was also associated with increased nitrate accumulation compared with the other lighting treatments. Nevertheless, nitrate concentrations remained within reported acceptable limits for lettuce production.

Estimated energy analysis demonstrated that LED2 required greater electrical energy input than LED1 and LED3; however, its higher productivity resulted in EUE values comparable to those observed under LED3 treatments. Slightly greater EUE values were obtained under LED3 combinations, suggesting a relatively balanced relationship between biomass production and estimated energy demand under mixed-spectrum lighting conditions. These findings highlight trade-offs among biomass production, quality characteristics, and estimated energy-use performance across different spectral and cultivation system combinations.

This study emphasizes the importance of coordinated optimization of LED lighting and cultivation system design for lettuce production in indoor vertical farming systems. However, because economic performance and detailed physiological mechanisms were not directly evaluated, practical recommendations regarding optimal production strategies should still be interpreted cautiously. Future studies integrating physiological, biochemical, and economic analyses together with direct electrical measurements would further strengthen the understanding and optimization of such systems.

## Data Availability

All data generated or analyzed during this study are included in this published article.
